# Supplementary material for: Reshuffling yeast chromosomes with CRISPR/Cas9
Source: PLoS Genet. 2019 Aug 29;15(8):e1008332. doi: 10.1371/journal.pgen.1008332 (PMC6738639; doi:10.1371/journal.pgen.1008332)
Supplement: S1 Fig — The sequences corresponding to donor oligonucleotides are shown in bold. The two gRNA target sequences are highlighted in light blue and orange. PAM sequences are highlighted in dark blue and orange. (PDF) [file pgen.1008332.s001.pdf]

```
         V 5'  ATTGCGCTCTTTCCCGACGAGAGTAAATGGCGAGGATACGTTCTCTATGGAGGATGGCATAGGTGATGAAGATGAAGGAGAAGTACAGAACGCTGAAGTGAA
               ||||||||||||||||||||||||||||||||||||||||||||||||||
      Vt XV 5'  ATTGCGCTCTTTCCCGACGAGAGTAAATGGCGAGGATACGTTCTCTATGGATGTAGGAACATCAACATGCTCAATCTCAATCGTTAGCACATCACATTTTTC
                                                                 ||||||||||||||||||||||||||||||||||||||||||||||||||
        XV 5'  TGGAGAAGGGTAAATTTTTAATTTGGGATGTTTTACTTGAAGATTCTTTAGTGTAGGAACATCAACATGCTCAATCTCAATCGTTAGCACATCACATTTTTC

        XV 5'  TGGAGAAGGGTAAATTTTTAATTTGGGATGTTTTACTTGAAGATTCTTTAGTGTAGGAACATCAACATGCTCAATCTCAATCGTTAGCACATCACATTTTTC
               ||||||||||||||||||||||||||||||||||||||||||||||||||
      XVt V 5'  TGGAGAAGGGTAAATTTTTAATTTGGGATGTTTTACTTGAAGATTCTTTAGGGATGGCATAGGTGATGAAGATGAAGGAGAAGTACAGAACGCTGAAGTGAA
                                                                 ||||||||||||||||||||||||||||||||||||||||||||||||||
         V 5'  ATTGCGCTCTTTCCCGACGAGAGTAAATGGCGAGGATACGTTCTCTATGGAGGATGGCATAGGTGATGAAGATGAAGGAGAAGTACAGAACGCTGAAGTGAA

         V   5'  ATTGCGCTCTTTCCCGACGAGAGTAAATGGCGAGGATACGTTCTCTATGGAGGATGGCATAGGTGATGAAGATGAAGGAGAAGTACAGAACGCTGAAGTGAA
                 ||||||||||||||||||||||||||||||||||||||||||||||||||||||||||||||||||||||||||||||||||||||||||||||||||||
restored V   5'  ATTGCGCTCTTTCCCGACGAGAGTAAATGGCGAGGATACGTTCTCTATGGAGGATGGCATAGGTGATGAAGATGAAGGAGAAGTACAGAACGCTGAAGTGAA

        XV  5'  TGGAGAAGGGTAAATTTTTAATTTGGGATGTTTTACTTGAAGATTCTTTAGTGTAGGAACATCAACATGCTCAATCTCAATCGTTAGCACATCACATTTTTC
                ||||||||||||||||||||||||||||||||||||||||||||||||||||||||||||||||||||||||||||||||||||||||||||||||||||
restored XV 5'  TGGAGAAGGGTAAATTTTTAATTTGGGATGTTTTACTTGAAGATTCTTTAGTGTAGGAACATCAACATGCTCAATCTCAATCGTTAGCACATCACATTTTTC
```

Supplementary figure 1
