# Supplementary figures and images for: Reshuffling yeast chromosomes with CRISPR/Cas9
Source: PLoS Genet. 2019 Aug 29;15(8):e1008332. doi: 10.1371/journal.pgen.1008332 (PMC6738639; doi:10.1371/journal.pgen.1008332)

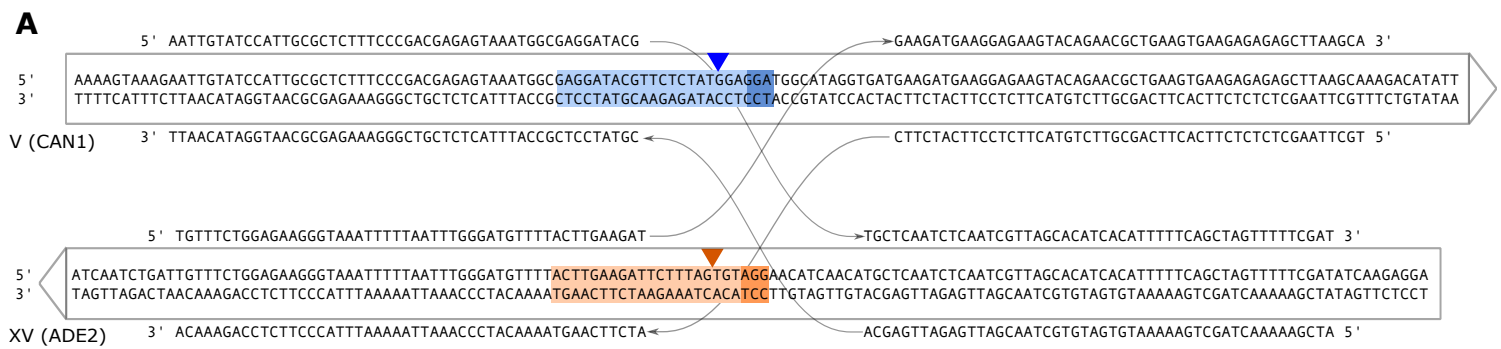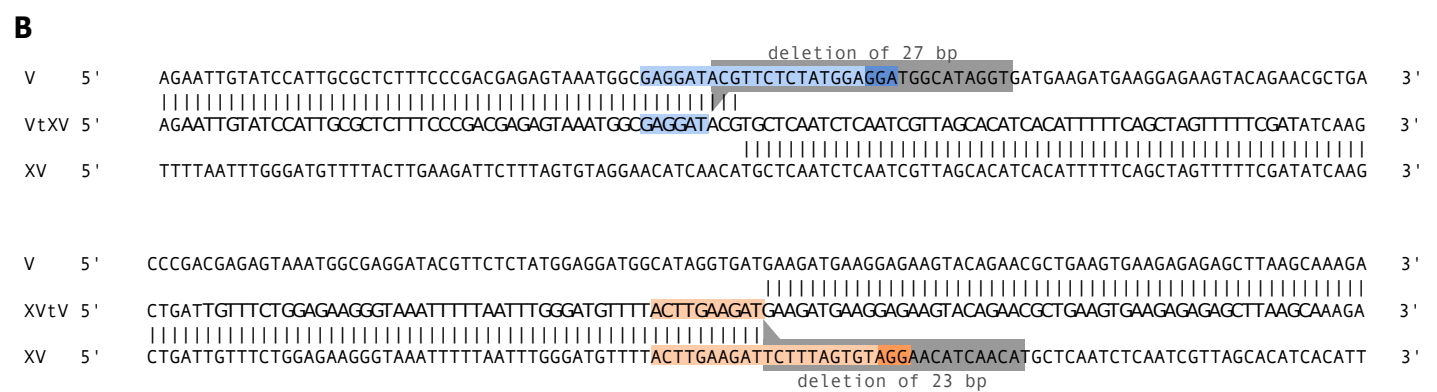

Supplementary figure 2

Supplement: S2 Fig — A. Targeted sequences and donors used to engineer the translocation with a small deletion. The two gRNA target sequences are highlighted in light blue and orange. PAM sequences are highlighted in dark blue and orange. Triangles indicate DSBs sites. Arrows framing the sequences indicate the orientation of coding phases. Donor nucleotides are represented above and below the frames by sequences linked by thin arrows to indicate their homology with the two different chromosomes. B. Alignments of the de novo assembled chimerical junctions on reference chromosomes V and XV. Donor sequences used to direct the translocation are in bold. Deleted sequences in chromosomes V and XV are highlighted in grey. The translocation occurred at the targeted position with a base-pair resolution. (PDF) [file pgen.1008332.s002.pdf]

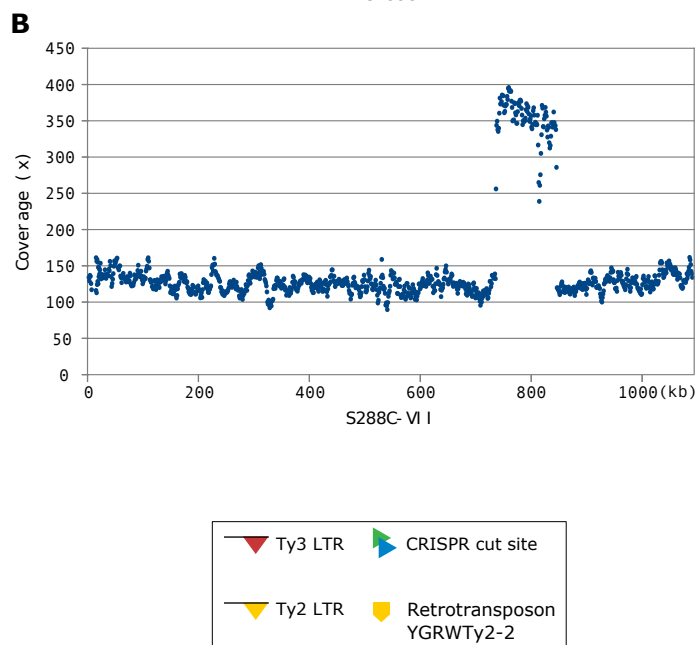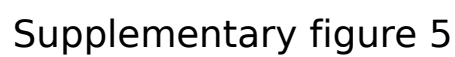

Supplement: S5 Fig — A. Homology matrix between the genomes of the strain showing an increase in global DNA content in PFGE (YAF064) and S288c. Translocated fragments are indicated by black arrows. The tandem triplication at the junction of chromosome VIItXV is indicated by the white arrow. B. Coverage of the YAF064 reads remapped on the reference chromosome VII. Each dot represents a window of 1 kb. C. Architecture of chromosomes VII (in green) and XV (in blue) of the reference strain and chimerical chromosomes VIItXV and XVtVII of the rearranged strain YAF064. Light grey triangles represent zoom-ins on chromosomal junctions. Ty3 LTRs and Ty2 LTRs elements are represented by red and yellow flags respectively. Full-length Ty2 elements are represented by yellow boxes. The Ty3 LTR copies targeted by CRISPR/Cas9 are indicated by green and blue triangles next to chromosomes VII and XV, respectively. The displaced 30 kb segment is referred to as region a and the other 80 kb segment as region b. Regions a and b, triplicated in the shuffled strain, are represented in lighter green shades. In summary, one copy of region a lies at the chimerical junction of chromosome XVtVII, whereas the remaining two and three copies of region a and b, respectively, are found in tandem at the chimerical junction of chromosome VIItXV. (PDF) [file pgen.1008332.s005.pdf]

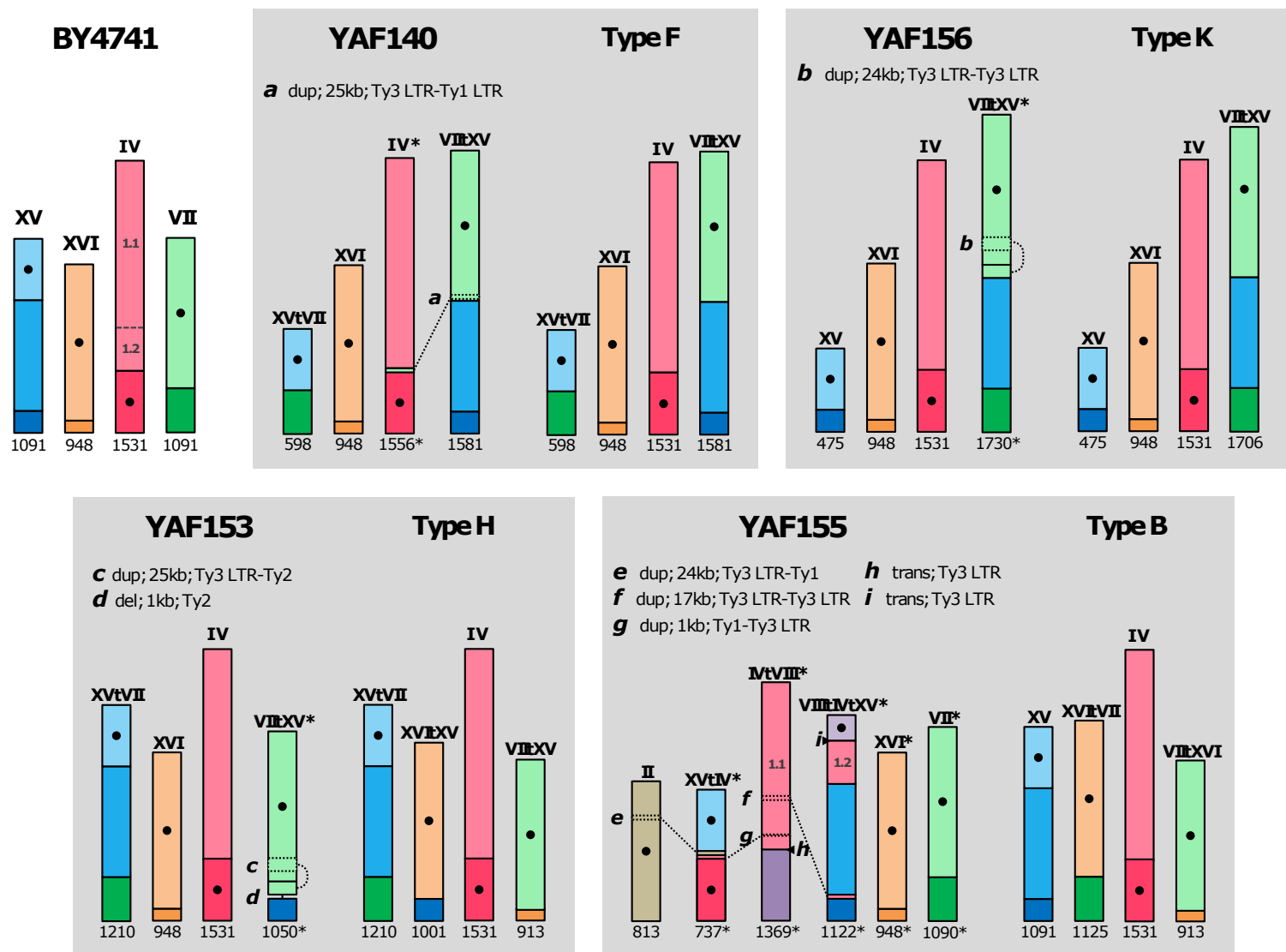

Supplementary figure 6

Supplement: S6 Fig — The wild type structure in BY4741 shows the 4 targeted chromosomes with black dots for centromeres. Each chromosome is fragmented by black lines representing DSB targeted Ty3 LTRs. Within chromosome IV a grey dotted line and sections named 1.1 and 1.2 represent an unpredicted position used for a reciprocal translocation in YAF155. Each shaded block contains both the chromosomal structure discovered using long read sequencing and the karyotype predicted by the CHEF gels (Type) for a single YAF strain. Below each chromosome is the size in kb. Stars on the chromosome name and size represent deviations from the corresponding karyotype predictions. Lower case italicized letters (a-i) represent unanticipated SVs captured by long reads. The key denotes from left to right, the type of SV (dup = duplication, del = deletion, trans = translocation), the size in kb and the repetitive element associated at the border of the element. For translocations only the SV type and repetitive element associated with the event is noted. For duplications, dotted lines represent the region duplicated and its new position. The SV d represents a deletion of 1 kb from XV followed by recombination within full length Ty2 elements. For YAF155 two additional chromosomes, II (brown) and VIII (purple), were involved. (PDF) [file pgen.1008332.s006.pdf]

Ratio to BY4741

All strains

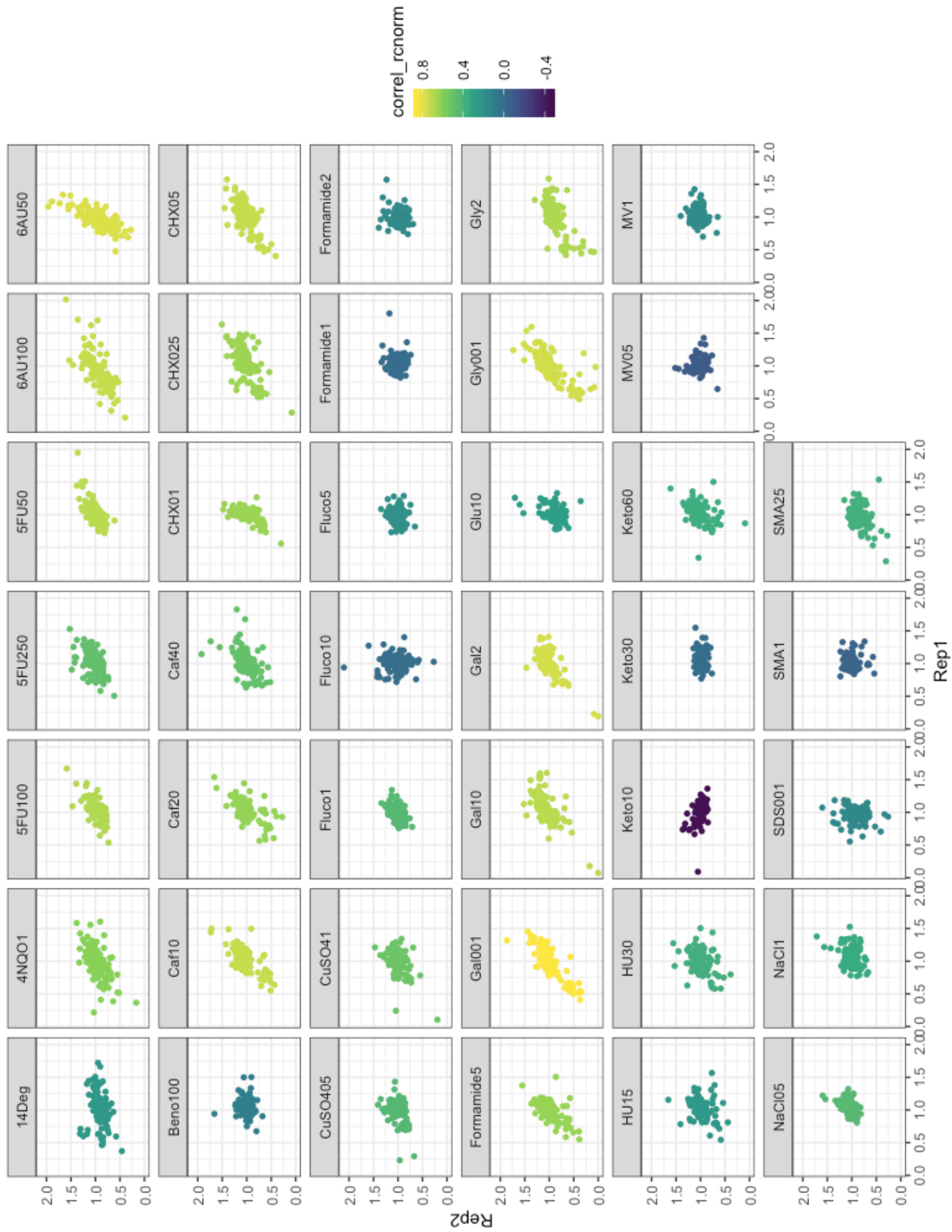

Supplementary figure 7

Supplement: S7 Fig — Each plot represents one condition and each dot represents the growth ratio of each strain (i.e. the colony size on the tested conditions divided by its size on SC) divided by the growth ration of BY4741. (PDF) [file pgen.1008332.s007.pdf]
