# Supplementary material for: Reshuffling yeast chromosomes with CRISPR/Cas9
Source: PLoS Genet. 2019 Aug 29;15(8):e1008332. doi: 10.1371/journal.pgen.1008332 (PMC6738639; doi:10.1371/journal.pgen.1008332)
Supplement: S3 Fig — A. gRNAs and donors used to perform the translocation between the promoters of SSU1 and ECM34. The micro-homology regions between the two chromosomes are highlighted in grey. The two gRNA target sequences are highlighted in light blue and orange. PAM sequences are highlighted in dark blue and orange. Triangles indicate DSBs sites. Mismatches brought by the donors to inactivate the PAMs are highlighted in black. B. Validation of the SSU1-ECM34 translocation by Southern blot. Left: schematic view of the chromosomal regions surrounding the translocation breakpoints. The double arrows indicate the length of the EcoRI restriction fragments. Right: Southern blot on the translocated strain YAF082 and parental strain BY4741. The probes are indicated on the top of the lanes. C. Sanger sequencing of YAF82 chimerical junctions. The two mismatches brought by the donors to prevent CRISPR/Cas9 activity after repair (indicated by stars) are present in chromosome XVItVIII. D. Multiple alignment of the promoter regions in front of the SSU1 gene in Y9J and YAF158 and of ECM34 in BY4741. Note that there are only 3 copies of the repeats present in the published assembly of the Y9J strain while it actually contains 4 repeats because the PCR product inserted in the YAF158 strain was amplified using the Y9J genome as matrix. There is only 1 copy of the 76 repeat region in the BY4741 genome. The 2 black arrows point to one indel and 1 SNP between the sequence of the BY4741 and the 2 other strains. (PDF) [file pgen.1008332.s003.pdf]

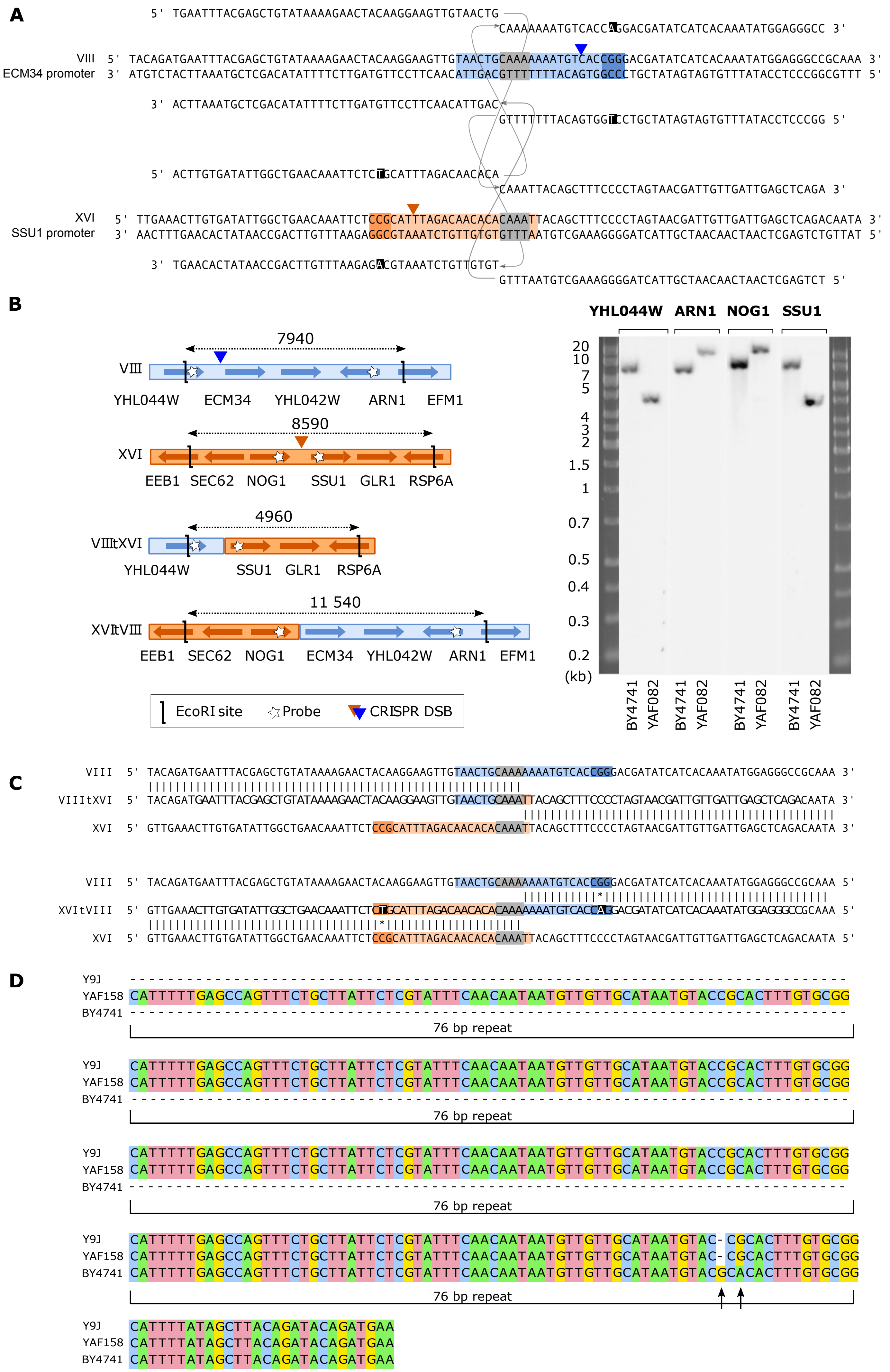

Supplementary figure 3
