# Supplementary material for: Reshuffling yeast chromosomes with CRISPR/Cas9
Source: PLoS Genet. 2019 Aug 29;15(8):e1008332. doi: 10.1371/journal.pgen.1008332 (PMC6738639; doi:10.1371/journal.pgen.1008332)
Supplement: S4 Fig — Junctions where the PAM was mutated are indicated by white triangles. Sequences where the PAM is intact are indicated by black triangles and PAM is highlighted in black. (PDF) [file pgen.1008332.s004.pdf]

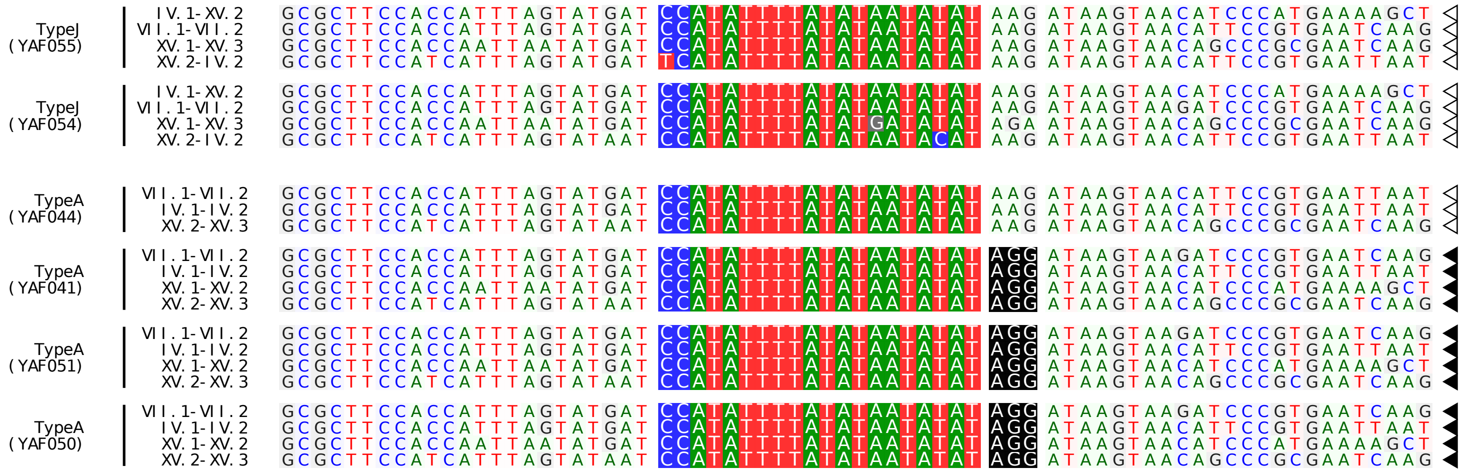


Supplementary figure 4
